# Multi-Lag Analysis of Symbolic Entropies on EEG Recordings for Distress Recognition

**DOI:** 10.3389/fninf.2019.00040

**Published:** 2019-06-04

**Authors:** Arturo Martínez-Rodrigo, Beatriz García-Martínez, Luciano Zunino, Raúl Alcaraz, Antonio Fernández-Caballero

**Affiliations:** ^1^Departamento de Sistemas Informáticos, Escuela Politécnica de Cuenca, Universidad de Castilla-La Mancha, Cuenca, Spain; ^2^Instituto de Tecnologías Audiovisuales de Castilla-La Mancha, Universidad de Castilla-La Mancha, Cuenca, Spain; ^3^Departamento de Sistemas Informáticos, Escuela Técnica Superior de Ingenieros Industriales, Universidad de Castilla-La Mancha, Albacete, Spain; ^4^Instituto de Investigación en Informática de Albacete, Universidad de Castilla-La Mancha, Albacete, Spain; ^5^Centro de Investigaciones Ópticas (CONICET La Plata–CIC), C.C. 3, Gonnet, Argentina; ^6^Departamento de Ciencias Básicas, Facultad de Ingeniería, Universidad Nacional de La Plata, La Plata, Argentina; ^7^Research Group in Electronic, Biomedical and Telecommunication Engineering, Escuela Politécnica de Cuenca, Universidad de Castilla-La Mancha, Cuenca, Spain; ^8^CIBERSAM (Biomedical Research Networking Centre in Mental Health), Madrid, Spain

**Keywords:** electroencephalography, distress, non-linear metrics, delayed permutation entropy, permutation min-entropy

## Abstract

Distress is a critical problem in developed societies given its long-term negative effects on physical and mental health. The interest in studying this emotion has notably increased during last years, being electroencephalography (EEG) signals preferred over other physiological variables in this research field. In addition, the non-stationary nature of brain dynamics has impulsed the use of non-linear metrics, such as symbolic entropies in brain signal analysis. Thus, the influence of time-lag on brain patterns assessment has not been tested. Hence, in the present study two permutation entropies denominated Delayed Permutation Entropy and Permutation Min-Entropy have been computed for the first time at different time-lags to discern between emotional states of calmness and distress from EEG signals. Moreover, a number of curve-related features were also calculated to assess brain dynamics across different temporal intervals. Complementary information among these variables was studied through sequential forward selection and 10-fold cross-validation approaches. According to the results obtained, the multi-lag entropy analysis has been able to reveal new significant insights so far undiscovered, thus notably improving the process of distress recognition from EEG recordings.

## 1. Introduction

Emotions are essential in human communication and interaction, and considerably influence on daily tasks related to cognition, perception and rational decision-making processes (Coan and Allen, 2007). Traditional techniques for emotion recognition are mainly focused on the analysis of physical aspects like facial expressions and speech characteristics (Calvo and D'Mello, 2010). However, given that emotional responses are initiated in the brain and then spread to other biological systems (Gao et al., 2015), interest in electroencephalogram (EEG) signals for emotion recognition has notably increased during the last years (Martínez-Rodrigo et al., 2017; Fernández-Sotos et al., 2018; Ramirez et al., 2018).

Existing affect models include from a few basic emotions (Ekman, 1992) to a wide variety of emotional states derived from the combination of basic ones (Schröder and Cowie, 2011). Russell's circumplex affect model is one of the approaches most widely used for emotion classification (Russell, 1980). In this bidimensional approach, emotions are distributed according to their level of valence (ranging from negative to positive) and arousal (from deactivated to activated). A relevant emotion that is receiving growing attention is negative stress, also called distress, because it presents a high prevalence in developed countries (Bong et al., 2013; Alberdi et al., 2016). Although today short-term distress is not considered a risk factor for health, a chronic condition of this emotion often causes or aggravates physical and mental disorders (Bender and Alloy, 2011; Mozos et al., 2017). In this regard, automatic distress identification from EEG signals would help prevent health problems and improve people's quality of life.

Since neural processes are non-linear and non-stationary, both at cellular and global level (Cao et al., 2015), non-linear metrics applied to EEG signal analysis should provide more relevant findings than linear indices traditionally used (Valenza et al., 2012). But, few studies have applied non-linearity to automatic detection of negative stress through EEG recordings (García-Martínez et al., 2019a). This is the case of symbolic entropies, such as Permutation Entropy (PE) (Bandt and Pompe, 2002) and Amplitude-Aware Permutation Entropy (AAPE) (Azami and Escudero, 2016), having demonstrated their efficiency in discriminating between calmness and distress (Hosseini and Naghibi-Sistani, 2011; Garćıa-Mart́ınez et al., 2017; Martínez-Rodrigo et al., 2019). Here, the quantification of similar patterns is typically obtained through consecutive samples, or their averaging, within a complete time series.

No lag or time delay between patterns is necessary in those cases where the autocorrelation function of the signal presents a steep decay. However, a time series with long-range linear correlations shows a slow decay in its autocorrelation function. Not applying a lag may hinder entropy metrics from properly quantifying the complexity and non-linear dynamics of the signal. Indeed, it has already been demonstrated that time-delayed entropy tests are helpful to diminish the influence of autocorrelation for better evaluation of the non-linearity of time series (Kaffashi et al., 2008). Hence, a multi-lag approach has been applied to localization of epileptogenic areas through EEG recordings (Zhu et al., 2015).

Let us highlight that an improvement of PE called Permutation Min-Entropy (PME) has been recently introduced (Zunino et al., 2015). PME consists of an improved time-delayed symbolic alternative for identifying the existence of hidden temporal correlations in time series. This allows a better discrimination of time series with similar temporal correlations. Moreover, PME has been very recently applied to emotion recognition by using heart rate variability (Xia et al., 2018). The promising outcomes open a door to the hypothesis that time-delayed analysis may uncover existing information in physiological systems, not revealed before through non-delayed or basic multiscale entropy (MSE) analyses. Furthermore, to the best of our knowledge, no previous research has focused on the study of multi-lag approaches for emotion recognition from EEG signals.

For this reason, in the present manuscript a time-delayed version of AAPE—called Delayed Permutation Entropy (DPE)—and PME metrics are applied for the first time with several time delays for the sake of checking the influence of the lag on discrimination between calmness and distress from EEG recordings. The remainder of this paper is structured as follows. Section 2 details the analyzed database, the DPE and PME metrics computed from the EEG recordings and the statistical analysis. Section 3 summarizes the results, which are then discussed in section 4. Finally, section 5 concludes the most remarkable findings related to this study.

## 2. Materials and Methods

### 2.1. Database

EEG signals were extracted from the publicly available Database for Emotion Analysis using Physiological Signals (DEAP) (Koelstra et al., 2012) to guarantee the reproducibility of this study as well as its fair comparison with previous or future works. This dataset contains a total of 1,280 EEG recordings and other peripheral variables from 32 healthy participants with ages ranging 19–37 (mean age of 26.9; 50% male) under different affective conditions. Forty one-minute length video clips with emotional content were used as stimuli in the experiment leading to the dataset. After each visualization, the participants described their emotional state by means of self-assessment manikins (SAM), two graphical scales representing nine intensity levels of valence and arousal (Morris, 1995).

**Figure 1:**
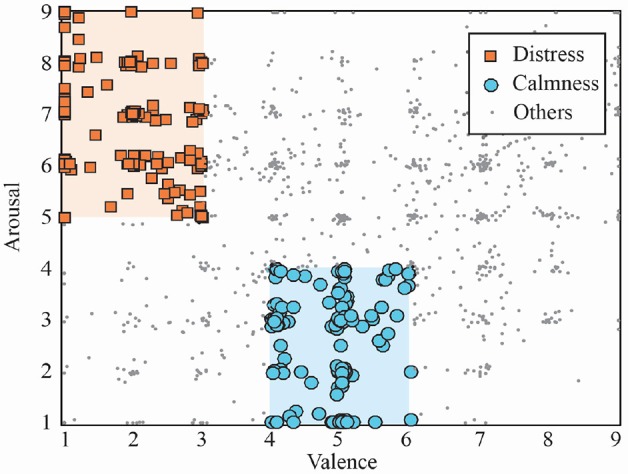
Trials distribution included in DEAP database in the arousal–valence space. Selected groups of distress and calmness trials are highlighted.

Although the trials contained within the dataset cover the whole valence-arousal space, only two subsets corresponding to distress and calmness emotional states were studied in the present study, as shown in Figure 1. Indeed, calmness and distress groups were selected according to previous works dealing with the same problem (Bastos Filho et al., 2012; Pomer-Escher et al., 2014; García-Martínez et al., 2016; Garćıa-Mart́ınez et al., 2017). Hence, distress trials were selected from arousal and valence levels higher than 5 and lower than 3, respectively. On the other hand, the calmness group contained trials with arousal and valence values lower than 4 and between 4 and 6, respectively. Therefore, a total number of 122 and 137 trials of distress and calmness, respectively, were finally analyzed in this work. Moreover, it is important to highlight that only the last 30 s of each trial were selected for further analysis.

### 2.2. EEG Signal Preprocessing

EEG signals were recorded at a sampling rate of 512 Hz with 32 electrodes placed according to the 10–20 standard system of electrode location (Klem et al., 1999). Before starting any kind of analysis, the recordings were preprocessed to eliminate noise and artifacts, thus preserving only the information related to brain activity. To this respect, the signals were initially down-sampled to 128 Hz and all EEG channels were re-referenced to the average potential of all electrodes. Next, a forward/backward high-pass filter at 3 Hz and a low-pass filter at 45 Hz cutoff frequency were applied to remove baseline and power line interferences, maintaining the frequency bands of interest in EEG recordings (Koelstra et al., 2012). After that, artifacts derived from physical activity (e.g., facial movements, eye blinks, heart bumping, etc.) and technical sources, such as electrode-pops were eliminated by means of independent component analysis (ICA) (Goh et al., 2017).

A well-known method with ability to automatically identify noisy independent components (ICs) was used (Nolan et al., 2010). Briefly, the algorithm firstly computed correlation between all ICs and electrooculography channels, as well as spatial kurtosis, power spectrum slope, Hurst exponent and median gradient for all ICs. Those components presenting at least an index with a value three times higher than standard deviation for all ICs were then removed. As a final step, the denoised EEG signal was reconstructed from the remaining ICs. It is worth noting that 1.05 ± 0.60 ICs were removed in average for each trial. More precisely, any artifactual IC was identified in 38 trials (14.67%), only one was removed in 168 trials (64.86%) and two were rejected in the remaining 53 trials (20.47%).

The EEG channels presenting high-amplitude noise were also detected and replaced by interpolation from adjacent electrodes (Reis et al., 2014). Although these signals were identified before ICA-based denoising of artifacts, their interpolation was developed after that preprocessing. This approach has been previously used by other authors (Forscher et al., 2016; Pincham et al., 2016; Bennett et al., 2018) and its main goal is to avoid mixing any non-linearity introduced by interpolation into the ICA decomposition (Nolan et al., 2010). Nonetheless, noisy EEG channels did not contribute to the rejection of artifacts (Nolan et al., 2010). As a result, the number of interpolated EEG channels was zero for 162 trials (62.55%), one for 83 trials (32.05%), two for 13 trials (5.02%) and three for the remaining trial (0.39%). Additionally, the most frequently interpolated channels were CP1 (in 21 trials, 21.65%), T8 (in 11 trials, 11.34%), CP5 (in 9 trial, 9.28%), AF4 (in 8 trial, 8.25%), T7 (in 6 trials, 6.19%), and FC2 (in 5 trials, 5.15%). The remaining channels were interpolated in <4% of trials.

### 2.3. Time-Delayed Version of Amplitude-Aware Permutation Entropy

Amplitude-Aware Permutation Entropy (AAPE) is an improvement of Permutation Entropy (PE) to consider amplitude information from analyzed time series (Fadlallah et al., 2013). Although this index has been mainly used in single-lag analyses, it can be adapted to deal with different time scales by changing the embedding delay τ (Azami and Escudero, 2016). Thus, for delayed-time PE computation, a time series *x*(*n*) = {*x*(1), *x*(2), …, *x*(*N*)} of length *N* is converted into *N* − (*m* − 1)·τ vectors of *m* samples, such that ${\text{X}}_{i,m}^{\tau }=\left\{X_{i,m}^{\tau }\left(1\right),X_{i,m}^{\tau }\left(2\right),\ldots ,X_{i,m}^{\tau }\left(m\right)\right\}=\left\{x\left(i\right),x\left(i+\tau \right)\ldots ,x\left(i+\left(m-1\right)\cdot \tau \right)\right\}$, for 1 ≤ *i* ≤ *N* − (*m* − 1)·τ. Each vector ${\text{X}}_{i,m}^{\tau }$ is associated with an ordinal pattern, described as permutation κ_*i*_ = {*r*_0_, *r*_1_, …, *r*_*m*−1_} of {0, 1, …, *m* − 1}, such that its single components fulfill $X_{i,m}^{\tau }\left(r_{0}\right)\leq X_{i,m}^{\tau }\left(r_{1}\right)\leq \ldots \leq X_{i,m}^{\tau }\left(r_{m-2}\right)\leq X_{i,m}^{\tau }\left(r_{m-1}\right)$. Hence, a total number of *m*! ordinal sequences π_*k*_ are obtained from patterns ${\text{X}}_{m}^{\tau }$. Then, the relative frequency of each sequence π_*k*_ is used to estimate its probability of appearance such that

(1) $$\begin{array}{r} p^{\tau }(\pi_{k})=\frac{\sum_{i=1}^{N-(m-1)\cdot \tau }\delta (\pi_{k},\kappa_{i})}{N-(m-1)\cdot \tau }, \end{array}$$

being δ(*u, v*) the Kronecker delta function modified specifically to work with sequences, i.e.,

(2) $$\begin{array}{l} \delta (u,v)=\{\begin{array}{ll} 1, & \text{if }u(i)=v(i),\text{for every }i=1,2,\ldots ,m;\\ 0, & \text{for otherwise}. \end{array} \end{array}$$

Then, delayed-time PE is finally obtained by computing the Shannon entropy from the probability distribution of all symbols, such that

(3) $$\begin{array}{r} \text{PE}(x,m,\tau )=-\frac{1}{\ln(m!)}\sum_{k=1}^{m!}p^{\tau }(\pi_{k})\cdot \ln\left(p^{\tau }(\pi_{k})\right). \end{array}$$

The index is normalized by term *ln*(*m*!) to obtain values ranging between 0 and 1. In the case of a completely predictable signal, only a pattern π_*k*_ is found and PE reports a 0 value. On the contrary, symbols π_*k*_ in unpredictable time series present the same probability of occurrence. Thus, PE provides the highest value 1. Hence, predictability information reported by PE is easily interpretable (Zanin et al., 2012). Nevertheless, only the ordinal structure of patterns is considered by this index, thus discarding the information related to the amplitude of each sample.

As amplitude differences could play a key role to determine the predictability of a time series, AAPE was introduced to overcome this limitation (Azami and Escudero, 2016). AAPE computation is based on calculating the probability of repetition of each pattern π_*k*_ by considering its relative frequency, and also the average absolute (AA) and relative amplitudes (RA) of vectors ${\text{X}}_{i,m}^{\tau }$. Amplitudes AA and RA are obtained, respectively, for a specific vector ${\text{X}}_{i,m}^{\tau }$ as

(4) $$\begin{array}{r} AA_{i}^{\tau }=\frac{1}{m}\sum_{l=1}^{m}|X_{i,m}^{\tau }(l)| \end{array}$$

and

(5) $$\begin{array}{r} RA_{i}^{\tau }=\frac{1}{m-1}\sum_{l=2}^{m}|X_{i,m}^{\tau }(l)-X_{i,m}^{\tau }(l-1)| \end{array}$$

Then, the relative frequency of π_*k*_ is computed as

(6) $$\begin{array}{r} p^{\tau *}(\pi_{k})=\frac{\sum_{i=1}^{N-(m-1)\cdot \tau }\delta (\pi_{k},\kappa_{i})\cdot \left(K\cdot AA_{i}^{\tau }+(1-K)\cdot RA_{i}^{\tau }\right)}{\sum_{i=1}^{N-(m-1)\cdot \tau }K\cdot AA_{i}^{\tau }+(1-K)\cdot RA_{i}^{\tau }}, \end{array}$$

being *K* an adjusting coefficient of terms AA and RA, ranging from 0 to 1. As recommended by the authors, a value *K* = 0.5 was considered here. Finally, delayed-time AAPE, referred to as Delayed Permutation Entropy (DPE), is computed by means of Shannon entropy, such that

(7) $$\begin{array}{r} \text{DPE}(x,m,\tau )=-\frac{1}{\ln(m!)}\sum_{k=1}^{m!}p^{\tau *}(\pi_{k})\cdot \ln\left(p^{\tau *}(\pi_{k})\right). \end{array}$$

### 2.4. Permutation Min-Entropy

Recently, PE has also been generalized by replacing Shannon entropy with Rényi one, reaching a better characterization of some rare and frequent ordinal patterns (Zhao et al., 2013). More precisely, Rényi Permutation Entropy (RPE) is defined as

(8) $$\begin{array}{r} \text{RPE}(x,m,\tau ,q)=\frac{1}{\ln(m!)}\cdot \frac{1}{1-q}\cdot \ln\left(\sum_{k=1}^{m!}p^{\tau }{\left(\pi_{k}\right)}^{q}\right), \end{array}$$

where order *q* (*q* ≥ 0 and *q* ≠ 1) is a bias parameter. Indeed, *q* < 1 benefits rare events and, contrarily, *q* > 1 privileges salient ones. It is mandatory to note that Shannon entropy is an instance of Rényi entropy for *q* = 1 and, hence, RPE is a more flexible tool than PE. In this respect, RPE has reported a more complete characterization of a variety of complex dynamics, including physiological processes (Mammone et al., 2015). In addition, RPE is featured to converge to a minimum entropy in the limit *q* → ∞, thus providing Permutation Min-Entropy (PME) (Zunino et al., 2015). This new entropy-based metric is quickly and simply computed as

(9) $$\begin{array}{r} \text{PME}(x,m,\tau )=-\frac{1}{\ln(m!)}\ln\left(\underset{{}_{k=1,2,\ldots ,m!}}{\max}[p^{\tau }\left(\pi_{k}\right)]\right), \end{array}$$

still retaining the main advantages of PE, such as its simplicity, low computational cost, noise robustness, and invariance with respect to non-linear monotonous transformations. This index has also proven a greater ability than PE to detect the existence of subtle temporal structures in EEG channels (Zunino et al., 2015).

### 2.5. Feature Selection

Firstly, single DPE and PME values from lag τ = 1 to lag τ = 10 were computed for each subject by using a pattern length *m* = 6. Entropies computed for each time-lag are a measure of predictability of the time series and assess the effect of time dynamics from an inter-lag perspective. Indeed, larger entropy values represent more unpredictable dynamics of the EEG signals, showing an increase of autocorrelated patterns in a long-term fashion.

On the other hand, multi-lag entropy curves were parameterized by means of slopes, areas under curves and arc lengths. Indeed, some studies have previously reported that features extracted from parameterized curves may reveal important information related to the dynamics of the signals across different temporal intervals (Escudero et al., 2006). In this regard, to estimate the trend evolution of each time-lag curve, slopes between delay τ = 1 and τ = 2, 4, 6, 8, and 10 were calculated from all EEG channels of each trial and denoted as *Slp*1 − τ. The slope is estimated as the straight line connecting the multi-lag entropy values under study. Higher slope values suggest larger entropy increases between the original signal (τ = 1) and higher versions in consecutive multi-lag time delays (τ = 2–10).

Furthermore, areas enclosed under the multi-lag curve between lag τ = 1 and lags τ = 2, τ = 4, τ = 6, τ = 8, and τ = 10 were computed and denoted as *Ar*1 − τ. In this sense, a higher area is achieved when DPE and PME entropy values are higher for the majority of time delays, suggesting that time series are less predictable. Finally, the arc length (*AL*) for each time-delayed curve was computed between lags τ = 2 and τ = 10. An arc length value shows the morphological alterations of the curve across different lags, and may show significant differences among lags from different groups of study. The arc length of each multi-lag curve was computed as

(10) $$\begin{array}{r} AL=\sum_{\tau =2}^{\tau =10}\sqrt{1+{\left(E[\tau ]-E[\tau -1]\right)}^{2}} \end{array}$$

referring *E* to the values of DPE and PME for the corresponding time-lag τ in each case.

Hence, a total of 21 features were computed for symbolic-based entropies DPE and PME on each EEG channel. More precisely, 10 single entropy values (one for each of the 10 time-lags computed), 5 tendency parameters related to time-lag curves (slopes *Slp*1 − 2, *Slp*1 − 4, *Slp*1 − 6, *Slp*1 − 8, *Slp*1 − 10), and 6 shape-related features (areas under curves *Ar*1 − 2, *Ar*1 − 4, *Ar*1 − 6, *Ar*1 − 8, *Ar*1 − 10, and arc length *AL*) were obtained for each EEG channel.

### 2.6. Statistical Analysis

Once the features were computed for each metric under study, Shapiro-Wilks and Levene tests corroborated the normality and homoscedasticity of the data, such that the results are expressed as mean and standard deviation. Then, statistical differences between features obtained for emotional states of calmness and distress were assessed for each time lag τ using a one-way analysis of variance (ANOVA). A value of statistical significance ρ < 0.05 was considered as significant.

Furthermore, the discriminatory power of each feature to distinguish between both groups of emotions was tested by using a stratified 10-fold cross-validation scheme. This methodology prevents over-fitting as well as other biases when performing the training/test operation on classifiers (Jung and Hu, 2015). Thus, the database selection containing 259 recordings was sliced into ten equally-sized folds with a balanced number of trials from both groups. Next, ten iterations were performed, such that in each one 9 out of 10-folds were used as a training subset, and 1 out of 10-folds was used as the test subset. To perform the classification, a receiver operating characteristic (ROC) approach was computed using the training trials to obtain an optimal threshold, which was then used to classify the trials in the test subset. It is worth noting that the threshold was selected as the cut-off point that maximizes accuracy (Acc). Values of sensitivity (Se), specificity (Sp), and Acc, obtained from the 10 iterations, were finally averaged to provide global and robust estimates.

Keeping in mind the objective of assessing possible relationships and complementary information among features, several advanced classifiers were used. Thus, a decision tree classifier (DTC), a support vector machine (SVM), a quadratic discriminant analysis (QDA) and a k-nearest neighbor (KNN) classifier were used. Regarding DTC, the nodes' growth was stopped when each node solely contained either fragments from only one group or a number of trials <20% of the entire dataset. Moreover, every node was split by using an impurity-based Gini index. Furthermore, the SVM classifier was run with a cubic kernel function and kernel scale of 1. Finally, the KNN classifier used an Euclidean distance metric with 10 neighbors, where the weight of the distance was computed to perform the classification by means of squared inverse. Nonetheless, given the high amount of analyzed features (21 features × 2 metrics × 32 channels), the subset providing most information was selected in first place for each classifier. Thus, a sequential forward selection (SFS) approach was used to select the subset of features minimizing misclassification rate for each classifier. A stratified 10-fold cross-validation scheme was also used to reduce overfitting in this analysis.

## 3. Results

### 3.1. Results for Delayed Permutation Entropy and Permutation Min-Entropy

**Figure 2:**
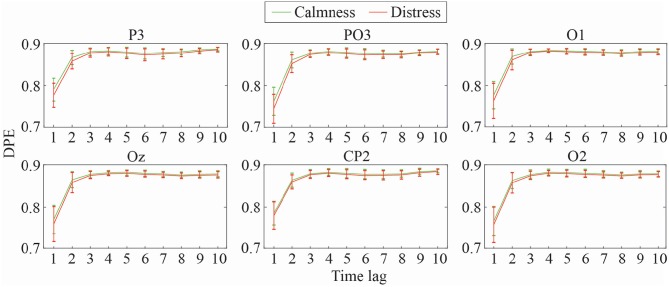
Mean and standard deviation values of DPE at different time-lags for calmness and distress at the most significant EEG channels.

**Figure 3:**
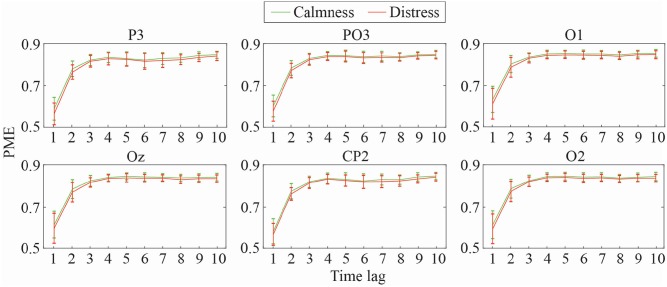
Mean and standard deviation values of PME at different time-lags for calmness and distress at the most significant EEG channels.

Mean and standard deviation of DPE and PME values for the most relevant EEG channels at different time-lags (1 ≤ τ ≤ 10) are shown in Figures 2, 3, respectively. As can be observed, both metrics obtained a similar trend throughout the increasing time-lags. DPE and PME values for calmness are higher than for distress trials, especially at lower lags. However, as time-lag increases the average differences between groups become smaller, such that at higher time-lags the mean entropy differences between groups become imperceptible. Furthermore, a certain degree of stabilization at time-lags >3 for both metrics can also be noticed, where the standard deviation decreases as the analyzed time-lag increases.

Table 1 shows the statistical significance and global classification performance, that is accuracy (Acc), for time-lags (1 ≤ τ ≤ 10) in DPE and PME metrics, respectively. Although there are similarities among mean entropy curves, the performance achieved for each metric differs considerably throughout the time-lags. In general terms, DPE shows a poorer performance discriminating between emotional states of calmness and distress than PME. As can be observed in Table 1, only lower time-lag entropies show a relevant statistical significance. Moreover, the overall discriminatory power for all cases is around 60%, decreasing even more when higher time-lags are analyzed. This effect is clearly seen at parieto-occipital and occipital channels PO3, O1, O2, and Oz. For instance, PO3 channel achieved a global performance of 62.9% at time-lag τ = 1, while accuracy decreased down to 57.10% at time-lag τ = 9. Only parietal channel P3 showed a regular statistical significance throughout every single time-lag, reaching a maximum global classification performance at time-lag τ = 2 with 63.7% of subjects classified correctly.

**Table 1:** Results of ρ and Acc of the most relevant EEG channels for DPE and PME at different time-lags.

**DPE**		**P3**	**PO3**	**O1**	**Oz**	**CP2**	**O2**
τ = 1	ρ	0.00261	8.85 × 10^−5^	0.0044	0.0235	0.132	0.0696
	Acc (%)	62.20	62.90	58.00	58.70	54.00	56.40
τ = 2	ρ	1.88 × 10^−4^	0.0049	0.0012	0.0139	0.0455	0.0436
	Acc (%)	63.70	60.89	60.62	61.78	61.00	61.39
τ = 3	ρ	3.23 × 10^−5^	0.913	0.0440	0.3023	0.3045	0.0436
	Acc (%)	62.50	56.00	58.80	57.10	54.40	53.30
τ = 4	ρ	6.08 × 10^−5^	0.4390	0.2140	0.6010	0.4710	0.6320
	Acc (%)	61.80	55.60	55.60	52.50	57.10	53.70
τ = 5	ρ	6.99 × 10^−6^	0.7310	0.0866	0.5114	0.2740	0.7310
	Acc (%)	61.40	59.85	61.00	64.09	61.00	62.16
τ = 6	ρ	2.69 × 10^−5^	0.1210	0.0312	0.341	0.697	0.45
	Acc (%)	62.20	59.80	57.60	56.80	55.40	53.30
τ = 7	ρ	3.76 × 10^−5^	0.0136	0.0278	0.28	0.185	0.4514
	Acc (%)	62.90	60.60	61.00	53.70	57.50	54.40
τ = 8	ρ	6.55 × 10^−5^	0.3520	0.0197	0.1130	0.1630	0.205
	Acc (%)	62.90	58.70	56.40	56.00	55.20	53.70
τ = 9	ρ	1.14 × 10^−4^	0.0932	0.0543	0.3210	0.0148	0.5670
	Acc (%)	61.00	57.10	55.00	55.60	60.20	54.80
**PME**		**P3**	**PO3**	**O1**	**Oz**	**CP2**	**O2**
τ = 1	ρ	3.33 × 10^−4^	8.85 × 10^−4^	0.017	0.128	0.0824	0.109
	Acc (%)	61.00	59.10	59.10	56.80	54.80	55.60
τ = 2	ρ	4.34 × 10^−4^	0.0049	0.0044	0.0637	0.0089	0.1610
	Acc (%)	61.40	59.10	61.00	56.80	56.50	56.40
τ = 3	ρ	0.0905	0.913	0.0532	0.362	0.342	0.633
	Acc (%)	59.80	60.50	59.80	58.30	57.10	53.70
τ = 4	ρ	0.034	0.439	0.865	0.169	0.476	0.764
	Acc (%)	59.20	56.00	56.80	57.80	59.80	61.70
τ = 5	ρ	0.379	0.731	0.181	0.166	0.346	0.749
	Acc (%)	57.50	58.70	64.50	61.00	62.50	60.20
τ = 6	ρ	0.216	0.121	0.0334	0.472	0.674	0.593
	Acc (%)	56.80	62.20	62.20	62.50	56.40	61.40
τ = 7	ρ	6.85 × 10^−4^	0.0136	0.194	0.974	0.0173	0.941
	Acc (%)	61.00	64.10	62.20	60.20	62.60	58.2
τ = 8	ρ	1.44 × 10^−4^	0.352	0.0786	0.0857	0.145	0.734
	Acc (%)	65.60	62.50	66.40	65.10	60.20	55.20
τ = 9	ρ	5.38 × 10^−5^	0.0932	0.0621	0.675	0.0026	0.798
	Acc (%)	68.30	62.90	60.20	59.10	68.70	58.70

On the contrary, PME showed better global classification performance for certain channels, especially at higher time-lags. For instance, parietal channel P3, classified correctly 68.3% of trials between calmness and distress at time-lag τ = 9. Similarly, centro-parietal channel CP2 showed a poor performance when no lag was applied (τ = 1), but it raised at higher time-lags, achieving the best single global performance at time-lag τ = 9, classifying correctly the 68.7% of the subjects. This improvement supposes an increase of more than 13% regarding PME at no lag τ = 1 and more than 8% compared with DPE metric at time-lag τ = 9. Moreover, there seems to be a certain degree of complementarity between DPE and PME at different time-lags, because the same EEG channels measured with each metric show relevant information at different time-lags. This contrast is well-noticed at parietal channel P3. No relevant differences between several time-lags were found when DPE was computed for P3, i.e., all time-lags presented a similar discriminatory power. On the contrary, the same channel showed an important increasing performance when it was analyzed by means of PME metric at higher time-lags; hence relevant information was noticed when time-delay was performed.

### 3.2. Results From Curve-Related Parameters

Table 2 summarizes diagnostic accuracy of every curve-based parameter derived from DPE analysis for the most relevant channels. As can be seen, almost all features achieved statistically significant differences between groups (ρ < 0.05). Features obtained from parietal channel P3 achieved a notable statistical significance, especially in the area under the time-lag curve (*Ar*1 − 2 to *Ar*1 − 6), thus stating the differences between curves at lower and their convergence at higher time-lags. It is also remarkable that parieto-occipital channel PO3 achieved a good performance for all features. With respect to slope-based parameters, global accuracy ranged from 60.62 to 62.23%, *Slp*1 − 10 being the feature with maximum performance for this channel. Similarly, accuracy of area-based lag parameters ranged from 59.07 to 62.16%, where *Ar*1 − 2 reported the maximal performance. It is also worth noting that arc-length reached the maximum global accuracy, classifying correctly 63.47% of trials, and thus overcoming the best performance obtained by single DPE entropy at the same channel at lag τ = 1. Finally, the rest of the parameters computed from occipital channels O1, O2, and Oz and centro-parietal channel CP2 obtained a more limited performance, their global accuracy ranging from 53.28 to 61.12%.

**Table 2:** Results of ρ and Acc of the DPE curve-related parameters.

**DPE**		**P3**	**PO3**	**O1**	**Oz**	**CP2**	**O2**
*Slp*1 − 2	ρ	0.0152	3.30 × 10^−4^	0.049	0.0970	0.5606	0.2044
	Acc (%)	57.92	61.78	57.14	57.92	54.05	58.69
*Slp*1 − 4	ρ	2.94 × 10^−4^	4.71 × 10^−5^	0.0048	0.0160	0.1783	0.0504
	Acc (%)	58.69	61.39	57.53	60.23	54.44	59.46
*Slp*1 − 6	ρ	4.60 × 10^−4^	6.92 × 10^−5^	0.0056	0.0189	0.1475	0.0576
	Acc (%)	58.30	60.62	59.46	61.12	53.28	58.30
*Slp*1 − 8	ρ	0.0011	1.78 × 10^−4^	0.0087	0.0350	0.3408	0.0909
	Acc (%)	58.69	62.16	58.30	58.69	53.67	58.42
*Slp*1 − 10	ρ	5.65 × 10^−4^	1.22 × 10^−4^	0.0089	0.0456	0.3002	0.0933
	Acc (%)	59.85	62.23	59.07	57.14	53.67	57.53
*Ar*1 − 2	ρ	4.30 × 10^−5^	5.48 × 10^−5^	0.0024	0.0174	0.0713	0.0542
	Acc (%)	61.67	62.16	59.07	57.45	55.09	56.76
*Ar*1 − 4	ρ	1.33 × 10^−4^	2.84 × 10^−4^	0.0025	0.0279	0.0717	0.0976
	Acc (%)	61.00	59.07	57.92	57.53	55.73	54.05
*Ar*1 − 6	ρ	1.14 × 10^−5^	0.0010	0.0031	0.0457	0.1075	0.1443
	Acc (%)	60.62	59.46	58.87	56.51	56.76	54.44
*Ar*1 − 8	ρ	0.0016	0.0015	0.0034	0.0543	0.0929	0.1609
	Acc (%)	60.23	60.62	58.30	58.69	55.98	53.28
*Ar*1 − 10	ρ	6.42 × 10^−4^	9.30 × 10^−4^	0.0027	0.0488	0.0516	0.1559
	Acc (%)	60.87	59.85	55.20	54.83	57.14	53.02
*AL*	ρ	0.0021	1.04 × 10^−4^	0.0136	0.0351	0.1672	0.0808
	Acc (%)	56.37	63.47	57.53	57.92	55.21	54.95

Similarly, Table 3 summarizes discriminant ability of all curve-based parameters derived from PME analysis for the most relevant channels. In this case, both statistical significance and global accuracy are more limited than for DPE curve-related parameters. All features computed on parietal channel P3 and parieto-occipital channel PO3 resulted to be statistical significant. The global accuracy obtained for these channels ranged from 57 to 61%, thus achieving a worse performance than before. Moreover, only a few curve-related parameters from occipital channels O1, O2, and Oz showed statistical significance, and the global accuracy was below 60% for all the parameters. Finally, CP2 achieved the worst performance, where global accuracy was around 55%.

**Table 3:** Results of ρ and Acc of the PME curve-related parameters.

**PME**		**P3**	**PO3**	**O1**	**Oz**	**CP2**	**O2**
*Slp*1 − 2	ρ	0.0324	0.0012	0.2999	0.5982	0.7753	0.2025
	Acc (%)	57.92	59.70	54.83	54.83	52.90	54.05
*Slp*1 − 4	ρ	0.0057	2.94 × 10^−4^	0.0148	0.05	0.1168	0.1127
	Acc (%)	58.38	59.07	58.69	59.07	56.37	55.98
*Slp*1 − 6	ρ	0.0013	4.28 × 10^−4^	0.0447	0.1396	0.0939	0.1025
	Acc (%)	60.62	58.69	58.42	54.83	55.21	55.60
*Slp*1 − 8	ρ	0.0116	1.89 × 10^−4^	0.0344	0.2420	0.1974	0.0688
	Acc (%)	58.69	57.92	53.53	54.05	52.51	55.64
*Slp*1 − 10	ρ	0.0063	2.85 × 10^−4^	0.0516	0.1980	0.1914	0.1682
	Acc (%)	60.68	61.39	58.30	56.37	55.21	54.83
*Ar*1 − 2	ρ	2.06 × 10^−4^	2.55 × 10^−4^	0.0081	0.0878	0.0292	0.1169
	Acc (%)	58.69	59.61	59.07	54.05	51.74	52.90
*Ar*1 − 4	ρ	8.10 × 10^−4^	0.009	0.0085	0.1381	0.0447	0.2704
	Acc (%)	58.87	59.46	59.85	54.33	52.90	55.41
*Ar*1 − 6	ρ	0.0060	0.0276	0.0089	0.1538	0.0930	0.3011
	Acc (%)	59.07	57.63	58.78	55.60	54.44	54.44
*Ar*1 − 8	ρ	0.0051	0.0204	0.0094	0.1734	0.0748	0.3732
	Acc (%)	57.92	58.30	59.85	55.21	55.48	53.00
*Ar*1 − 10	ρ	0.0016	0.0193	0.0072	0.1666	0.0440	0.4302
	Acc (%)	60.05	57.92	59.15	55.73	55.21	54.44
*AL*	ρ	0.0097	5.00 × 10^−4^	0.0950	0.1582	0.5373	0.0987
	Acc (%)	59.82	58.69	56.16	53.67	54.44	58.69

### 3.3. Multi-Parametric Analysis and Advanced Classification

**Figure 4:**
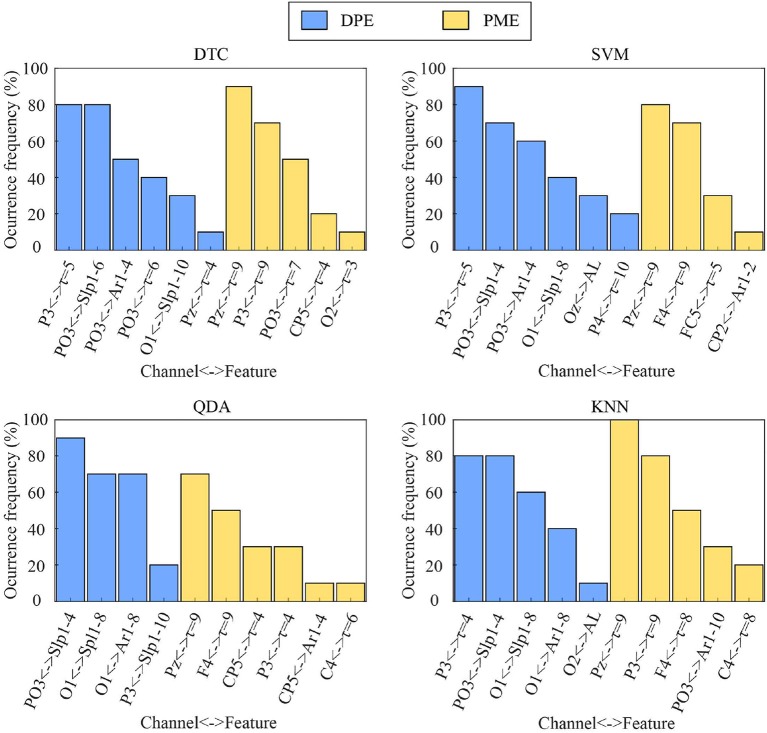
Occurrence of the most selected features through an SFS scheme within a 10-fold cross-validation approach for each classifier.

For each classifier, the optimal number of features minimizing its misclassification rate through an SFS scheme ranged from 5 to 8 in each iteration of a 10-fold cross-validation approach. The occurrence of the most relevant variables are displayed in Figure 4. As can be seen, entropy-based metrics were mainly chosen for time-lags longer than 1 and curve-related variables both from DPE and PME. Moreover, it should be noted that most of these features were selected in nearly all folds, thus only changing the less relevant ones for the resulting classification models. More precisely, for all classifiers, most DPE-based parameters were chosen from EEG channels P3 and PO3 and most PME-based features from channels P3, Pz, FC5, C4, and CP5.

Once the feature selection process finished for each classifier, the obtained classification results are displayed in Table 4 in terms of sensitivity, specificity and accuracy. Note that global discriminant ability for all approaches ranged from 75.66% (for QDA) to 92.32% (for KNN). Furthermore, the SVM classifier achieved a comparable performance to KNN, classifying correctly 91.12% trials. Finally, it should also be highlighted that both SVM and KNN classifiers reported the largest diagnostic accuracies with well-balanced values of sensitivity and specificity.

**Table 4:** Values of sensitivity, specificity, and accuracy obtained for each classifier once the feature selection process had finished.

	**DTC**	**SVM**	**QDA**	**KNN**
Sensitivity (%)	75.41	91.97	82.21	93.72
Specificity (%)	83.94	90.16	61.56	90.66
Accuracy (%)	79.92	91.12	75.66	92.32

## 4. Discussion

During the last years automatic emotion recognition has received special attention due to its importance in areas, such as medicine and education. Among the different types of emotions, continued distress is one of the most studied because it is often harmful for health. Considering its relevance, distress has been assessed in a wide variety of scenarios, including driving tasks (Healey and Picard, 2005), military exercises (Skinner and Simpson, 2002), surgical procedures (Marrelli et al., 2014), and on-line exams (Gomes et al., 2014), among others. An interesting study recently published shows a methodology to redirect stress episodes toward positive moods (Fernández-Caballero et al., 2016).

Taking into consideration this preamble, several works have been published in the literature. Their research is focused on automatic distress recognition using EEG recordings (Hosseini et al., 2010; Khosrowabadi et al., 2011; Peng et al., 2013; Minguillon et al., 2016; Al-Nafjan et al., 2017; Al-Shargie et al., 2018; Jebelli et al., 2018; Garćıa-Mart́ınez et al., 2019b). However, only a few of them have analyzed this phenomena from a non-linear point of view (García-Martínez et al., 2016). Recently, another approach reported that symbolic analysis of brain dynamics was able to detect distress (Garćıa-Mart́ınez et al., 2017). In that study, PE and its extension called AAPE were used to assess brain dynamics for each EEG channel. However, the analysis was carried out without considering the possibility of exploring hidden non-linear information at time-lags higher than one. This was the starting point that motivated the present study.

To the best of our knowledge, this is the first work addressing the effects of multi-lag for distress recognition from EEG recordings. For this purpose, a modified version of AAPE was used to analyze EEG signals with distinct time-lags. Additionally, PME was also considered in this study, since it is an improved symbolic alternative for identifying the existence of hidden temporal dynamics in time series and it allows a better discrimination of signals with similar temporal correlations (Zunino et al., 2015). Indeed, it has been recently applied in the study of emotion recognition using heart rate variability with promising results (Xia et al., 2018).

**Figure 5:**
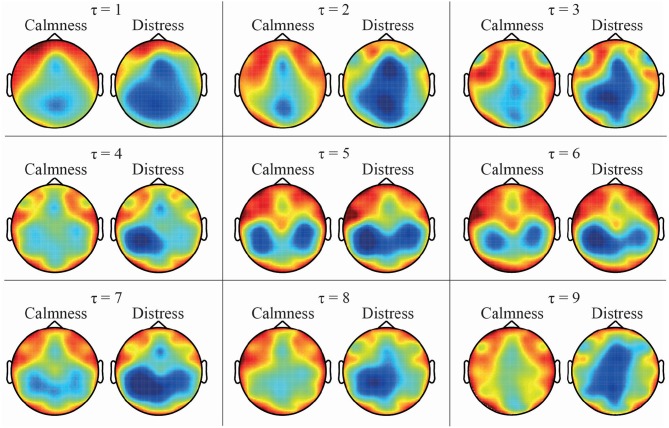
Topological EEG representation of average calmness and distress values for time-lags from τ = 1 to τ = 9 using PME metric.

As expected, both DPE and PME metrics reported the same trends when calculating the mean entropy values across the different time-lags, as was observed in Figures 2, 3. Calmness emotional state reported higher entropy values than distress for all EEG channels, especially at lower time-lags. However, this difference became smaller as the time-lag increased. Furthermore, the time-lag analysis revealed additional entropy information not observed at time-lag τ = 1, especially at centro-parietal and occipital channels. This effect can also be well-noticed in Figure 5, which shows a topological representation of mean entropy values for each channel at the first nine time-lags using PME. Although the general trend is maintained across time-lags, the imprint patterns change, thus revealing information at certain channels not seen before.

These results enhance the presence of a larger diversity of ordinal patterns in some local time series in calmness trials in comparison to distressed ones, thus suggesting the existence of more complex brain dynamics in calmness state. Such loss of complexity under distress might be associated to a lower brain's ability of adaptation to external stimuli and environmental changes. Indeed, decomplexification of physiological systems has been traditionally identified with a lower ability to manage information, and therefore with a higher probability of suffering a pathological condition (Goldberger et al., 2002; Lipsitz, 2004). Interestingly, these findings are in agreement with other studies published during the past years. In this sense, increased values of correlation dimension in calm participants with respect to distressed subjects have been reported so far (Hosseini et al., 2015). In another work, a decrease of relative power in subjects facing a series of distressful stimuli was also described (Bastos Filho et al., 2012). Finally, the fact that this trend is maintained throughout the different time-lags reinforces a previous study where no lag was applied (Garćıa-Mart́ınez et al., 2017).

Another relevant finding is that single discriminatory power in multi-lag analysis has notably improved with respect to other previous studies dealing with singe-lag (Garćıa-Mart́ınez et al., 2017) and MSE (Martínez-Rodrigo et al., 2019) analysis, especially in some specific channels. Thus, left parietal channel P3 is still a very relevant channel for distress detection using symbolic analysis. This finding was already reported in our recent previous work where AAPE was applied to the data (Garćıa-Mart́ınez et al., 2017). Nevertheless, other studies have already corroborated this association with the left parietal area. Thus, a higher activation has been observed during normal non-depressed and reasonably positive moods in the left parietal area than in the right one (Davidson, 1988). In the same line, meditation has also been characterized by an increasing activity of the left parietal region (Manna et al., 2010). In the present study, P3 showed robustness and consistency across different time-lags when discriminating between emotional states of calmness and distress. Nevertheless, the global classification performance was improved notably for time-lags higher than one, especially when data was analyzed by means of PME metric, increasing from 61% when no lag was applied up to 68.30% when time-lag τ = 9 was computed.

**Figure 6:**
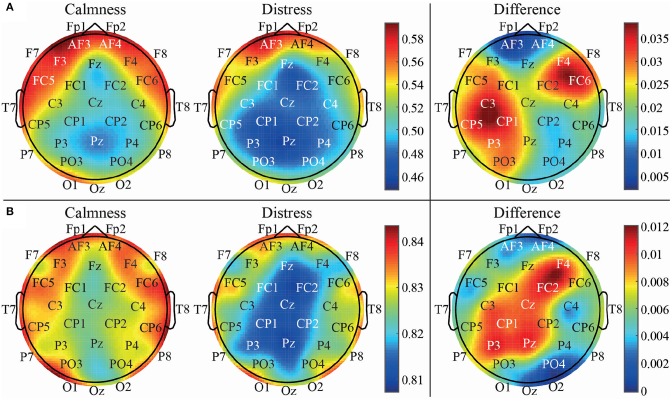
Topological EEG representation of average calm and distressed patients for time-lag τ = 1 vs. τ = 9 using PME metric.

These findings may indicate the existence of long-range correlations in the data, which have only been sufficiently highlighted by considering a multi-lag entropy-based analysis. Indeed, these observations can be visually corroborated in the topological representation of brain areas depicted in Figure 6. It represents average PME values computed from emotional states of calmness and distress for time-lags τ = 1 (a) and τ = 9 (b). As can be seen, entropy values obtained at time-lag τ = 9 are quite different compared to the analysis with no lag, showing a more balanced pattern between left and right hemispheres throughout frontal, parietal and occipital areas. In addition, the entropy differences between calmness and distress are also shown in this figure (on the right column). Thus, the higher differences are found in left central region for τ = 1, whereas a higher activation of left parietal region is obtained for τ = 9.

The right frontal channel F4 also presents a considerable difference of activation between calmness and distress states both in τ = 1 and τ = 9 cases. Interestingly, the relevance of the mentioned areas and the possible relation between frontal and parietal areas of opposite hemispheres has already been depicted in our previous studies. In fact, 30 years ago it was verified that a relative left parietal brain activation is balanced by a relative right parietal brain activation and vice-versa (Davidson, 1988). A similar outcome has also been observed in another study where patients with different mental disorders were conducted to practice meditation (Rubia, 2009).

Moreover, the right brain hemisphere deserves especial attention in this work. Considering our previous findings, the right centro-parietal channel CP2 showed no relevance when analyzing emotional states with symbolic entropies (Garćıa-Mart́ınez et al., 2017). This outcome has been corroborated again in this work, where neither DPE nor PME showed statistical significance at τ = 1 (no lag), and the global classification was below 55% in both cases (see Table 1). However, when analyzing the same brain area at higher time-lags, a notable increase of statistical significance and discriminatory power was observed. It is especially the case for CP2 with PME at time-lag τ = 9 (ρ = 0.0026 and Acc = 68.70%), thus achieving the highest global classification in this study. The relevance of the channel CP2 can also be observed in Figure 6, where the difference is not notable for τ = 1, but it is for τ = 9.

These findings, together with the relevance of the results reported by the left parietal channel P3, reveal the possible existence of complementary information among the parietal lobes of both brain hemispheres. Indeed, a number of previous works reported a certain degree of complementarity between right and left posterior areas under stimulation of distress and calmness. For instance, interesting information about parietal and occipital asymmetry at different frequency bands during distressful tasks has been described (Park et al., 2011). Furthermore, an intensive parietal lobe activation under anxiety and distress conditions has also been reported (Nitschke, 1998).

Recently, occipital electrodes O1 and O2 have also been explored to evaluate variations in complexity provoked by visual elicitation (Tonoyan et al., 2016). In a similar line, the posterior brain area has been related to the arousal component of emotions, thus being their processing essential for the recognition of emotions (Dolcos and Cabeza, 2002). Interestingly, in our previous study the combination of the left parietal channel P3 and the right parietal channel P4 achieved a notable performance discriminating between emotional states of calmness and distress, thus demonstrating the inter-correlation of these brain regions (Garćıa-Mart́ınez et al., 2017). However, in that study, brain dynamics were assessed by means of different computation approaches, where each methodology highlighted one of the hemispheres in isolation. In the present work, both areas have resulted to be significant when analyzed with PME metric at higher time-lags, obtaining results comparable to those reported individually by other metrics used in our previous works.

The obtained multi-lag curves were also parameterized and studied to compare the relative complexity of normalized time series. The use of curve profiles for characterization of biological signals has been already proposed by other authors (Costa et al., 2005). Accordingly, *Slp*1 − 4 and *Slp*1 − 6 reflect that the degree of change in the complexity of some EEG channels is more relevant in smaller time-lags. These outcomes were observed in Figures 2, 3, where changes in the slopes could be seen until the curves stabilized around time-lag τ = 5. In this regard, parietal and occipital channels showed statistical relevance at these slopes for DPE metric, but only parietal channels resulted relevant when curved-related parameters were calculated for PME metric. It is worth noting that the same outcomes were also obtained for the area under curve parameters.

On the other hand, the developed multivariate analysis has shown that putting all the data together led to a notably overall performance increase, which demonstrated that multi-lag analysis is able to provide additional, as well as complementary information, to single-lag one. To this respect, an SFS scheme was applied under a 10-fold cross-validation approach to choose the optimal subset of features maximizing the classification rate for each classifier. Interestingly, the most relevant features selected for each classifier were mainly computed from EEG channels showing the largest differences between emotional states of calmness and distress. Indeed, DPE computed from channels PO3 and P3 reported high statistically significant differences between the two groups of trials, as shown in Tables 1, 2. Similarly, PME obtained from channels P3, Pz, FC5, C4, and CP5 provided high visual differences, as shown in Figures 5, 6. Moreover, let us highlight that all selected features were computed from time-lag longer than 1 or from τ-based curves, as shown in Figure 4.

Another relevant aspect is that the results obtained in the present study outperformed notably other similar works that have analyzed non-linear metrics from the same database with no lag, such as summarized in Table 5. Indeed, a global accuracy of 69.6% has only been reported by applying a high-order crossing approach to four EEG channels (Bastos Filho et al., 2012). In addition, different non-linear metrics have reported a higher level of complexity in stressed subjects (Peng et al., 2013). In other work, combining quadratic sample entropy values from several EEG channels through a DTC classifier, a discriminant ability around 75% has been provided (García-Martínez et al., 2016). On the other hand, a discriminant model based on SVM and using irregularity and symbolic metrics reached a diagnostic accuracy >80% (Garćıa-Mart́ınez et al., 2019b). Similarly, variants of PE have already been applied to distress recognition, with a classification performance of 81.31% (Garćıa-Mart́ınez et al., 2017). However, in the present study an improvement of about 10% has been reported by making use of the same kind of SVM classifier.

**Table 5:** Comparison of the most relevant works dealing with automatic identification of negative stress from the recordings.

**Work**	**Experiment**	**Features**	**Statistics/Classifier**	**Results**
Hosseini et al., 2010	15 subjects	FD^b^, CD^c^, and WEn^d^	LDA^e^ and SVM	LDA: 80.1% SVM: 84.9%
	5 EEG channels			
	IAPS^a^			
Bastos Filho et al., 2012	32 subjects	Statistical features, PSD^f^, and HOC^g^	KNN	Stat.: 66.25%
	4 EEG channels			PSD: 70.1%
	Videoclips			HOC: 69.6%
Peng et al., 2013	13 subjects	CD, LZC^h^, LLE^i^, PSD	ANOVA	Higher complexity in stress
	3 EEG channels			
	Eyes closed, no stimuli			
García-Martínez et al., 2016	32 subjects	SE^j^, QSE^k^, and DE^l^	Decision tree	75.29%
	32 EEG channels			
	Videoclips			
Garćıa-Mart́ınez et al., 2017	32 subjects	QSE, PE, and AAPE	SVM	81.31%
	32 EEG channels			
	Videoclips			
Garćıa-Mart́ınez et al., 2019b	32 subjects	QSE, CE^m^, and CCE^n^	SVM	80.31%
	32 EEG channels			
	Videoclips			
This work	32 subjects	DPE and MPE	KNN	92.32%
	32 EEG channels			
	Videoclips			

a *IAPS, International Affective Picture System*.

b *FD, Fractal dimension*.

c *CD, Correlation dimension*.

d *WEn, Wavelet entropy*.

e *LDA, Linear discriminant analysis*.

f *PSD, Power spectral density*.

g *HOC, High-order crossings*.

h *LZC, Lempel-Ziv complexity*.

i *LLE, Largest Lyapunov exponent*.

j *SE, Sample entropy*.

k *QSE, Quadratic sample entropy*.

l *DE, Distribution entropy*.

m *CE, Conditional entropy*.

n *CCE, Corrected conditional entropy*.

Likewise, the classification results obtained in the present study also significantly improved the sole recent work conducting a MSE analysis on EEG signals for distress identification (Martínez-Rodrigo et al., 2019). In fact, making use of the same SVM-based approach, here a classification rate between distress and calmness emotional states has been obtained about 6% higher than for MSE. It should be noted that both MSE and multi-lag entropy analyses pursue the same goal of quantifying complexity at different time scales. For this purpose, MSE uses a rescaling procedure based on filtering out the shorter oscillations and keeping the longer ones (Humeau-Heurtier, 2015). This approach unavoidably removes some frequency content, specially from rescaled time series presenting very fast oscillations (Humeau-Heurtier, 2015). Such loss of frequency information could explain the aforementioned poorer outcome reached by MSE, because entropy computation from time-lagged samples does not alter time and frequency information from original data (Govindan et al., 2007; Kaffashi et al., 2008). Moreover, this finding could also justify the fact that, whereas no changes were noticed across all time scales in MSE analysis in brain areas activating and supporting distress (Martínez-Rodrigo et al., 2019), large differences have been observed for different time-lags, as extensively described in previous paragraphs.

Finally, there are some limitations in this study that deserve our attention. Firstly, the studied DEAP database is not exclusively designed for recognition of calmness and distress emotions. In fact, many other emotional states were also recorded during the experiment (Koelstra et al., 2012). Moreover, the number of trials eliciting calmness and distress is notably unbalanced for each healthy volunteer, thus making the use of a subject-based classification impossible. Secondly, further analyses on other similar databases like ASCERTAIN (Subramanian et al., 2018), AMIGOS (Miranda Correa et al., 2018), and DREAMER (Katsigiannis and Ramzan, 2018) are required to corroborate and generalize the obtained results. However, the impact of some potential confounding aspects on the results provided by several databases will have to be carefully analyzed for this purpose. Thus, it should be thoughtfully scrutinized how different experimental setups, population distributions in terms of age and gender, and technical aspects related to the acquisition of EEG signals mask changes in brain dynamics under distress. Thirdly, the video clips used as stimuli have a duration of 1 min, which may be too much time to just elicit a single emotional state. Thus, participants may present different emotions for the same stimulus, making it difficult to properly rate their level of valence and arousal. Finally, only EEG signals were assessed in this work, thus discarding the information reported by other physiological variables. However, peripheral recordings also contained in DEAP and other databases, in combination with brain dynamics, will be explored in further studies for the sake of detecting distress episodes.

## 5. Conclusions

In this study, two permutation entropies, adapted to work in a multi-lag context, have been analyzed for the first time to automatically identify negative stress. This multi-lag analysis has revealed new insights never seen before, thus notably improving the performance of distress identification. Considering the relevant results that permutation entropy has previously reported in non-lag and multiscale contexts for human emotion detection, it becomes highly interesting to analyze brain dynamics from a time delay viewpoint. For this reason, we hypothesized that there might exist relevant and complementary information at higher time-lags among different brain areas. The results obtained after performing the analyses have confirmed our initial ideas, reporting an improved classification between emotional states of calmness and distress.

Moreover, left parietal and right centro-parietal channels showed remarkable activation at higher time lags, suggesting that removing long-range linear correlations may help to better evaluate the non-linear information of the data. Finally, several discriminant models obtained from advanced classifiers were used to study the complementarity of the features computed at different time-lags for each EEG channel. The resulting functions have combined single entropy values from different channels calculated at lags higher than one with curve-related parameters, thus corroborating that there are more relevant information when time-lags are applied to the time series than when data are analyzed without any time delay or averaging consecutive samples.

## Author Contributions

AM-R and BG-M conceived and designed the study, programmed the experiments and drafted the manuscript. LZ and RA helped to interpret the results and reviewed the manuscript. Finally, AF-C supervised the experiments, reviewed the manuscript and contributed to the final version. All authors have read and approved the final version of the manuscript.

### Conflict of Interest Statement

The authors declare that the research was conducted in the absence of any commercial or financial relationships that could be construed as a potential conflict of interest.
